# Antibodies against malondialdehyde among 60-year-olds: prediction of cardiovascular disease

**DOI:** 10.1038/s41598-023-42264-1

**Published:** 2023-09-11

**Authors:** Shailesh Kumar Samal, Karin Leander, Max Vikström, Lena Griesbaum, Ulf de Faire, Johan Frostegård

**Affiliations:** 1 Unit of Immunology and Chronic Disease, Institute of Environmental Medicine, Karolinska Institutet, Nobels Väg 13, 17165 Stockholm, Sweden; 2 Division of Cardiovascular Epidemiology, Institute of Environmental Medicine, Karolinska Institutet, Stockholm, Sweden

**Keywords:** Immunology, Innate immunity, Pattern recognition receptors

## Abstract

Malondialdehyde (MDA) is generated in oxidized LDL. It forms covalent protein adducts, and is recognized by antibodies (anti-MDA). We previously studied IgM anti-MDA, and here we focus on IgG, IgG1 and IgG2 anti-MDA in predicting cardiovascular disease (CVD). We determined, by ELISA, anti-MDA in a 7-year follow-up of 60-year-old men and women from Stockholm County (2039 men, 2193 women). We identified 210 incident CVD cases (defined as new events of myocardial infarction (MI), and hospitalization for angina pectoris) and ischemic stroke, and 620 age- and sex-matched controls. IgG anti-MDA was not associated with CVD. Median values only differed significantly for IgG1 anti-MDA among men, with lower levels among cases than controls (p = 0.039). High IgG1 anti-MDA (above 75th percentile) was inversely associated with CVD risk after adjustment for smoking, body mass index, type 2 diabetes, hyperlipidemia, and hypertension, (OR and 95% CI: 0.59; 0.40–0.89). After stratification by sex, this association emerged in men (OR and 95% CI: 0.46; 0.27–0.77), but not in women. IgG2 anti-MDA were associated with protection in the whole group and among men though weaker than IgG1 anti-MDA. IgG2 anti-MDA above the 75th percentile was associated with an increased risk of MI/angina in women (OR and 95% CI: 2.57; (1.08–6.16)). IgG1 and less so IgG2 anti-MDA are protection markers for CVD and MI/angina in the whole group and among men. However, IgG2 anti-MDA was a risk marker for MI/angina among women. These findings could have implications for both prediction and therapy.

## Introduction

Atherosclerosis is characterized by accumulated dead cells and oxidized low-density lipoprotein (OxLDL) in the artery wall. This disease condition could therefore be described as a faltering clearance of these compounds. Typical atherosclerosis also involves activated immune competent cells, which produce cytokines, mainly pro-inflammatory. Since atherosclerosis is the main cause of the cardiovascular disease (CVD), the lack of clearance of dead cells and oxLDL thus contributes to the leading cause of death and morbidity worldwide. Instead, macrophages accumulate OxLDL and turn into inert foam cells, which, instead of transporting away their obnoxious load accumulate in the lesions and eventually die there^1, 2^.

OxLDL is immunogenic and antibodies against OxLDL are present at high levels in humans. However, their role has been debated and is not clear, since some publications reported anti-OxLDL being a risk marker. In contrast, we reported for the first time that antibodies, in this case anti-OxLDL can be associated with protection in borderline hypertension^3^. It is therefore of interest to investigate which antigens in the complex compound OxLDL play a role in disease development. Both malondialdehyde (MDA) and phosphorylcholine (PC) are generated during lipid peroxidation as in oxLDL, and both could be of interest in atherosclerosis pathogenesis. MDA and PC are danger associated molecular patterns (DAMPs) while PC is also a pathogen-associated molecular pattern, present in many bacteria. Both anti-MDA and anti-PC have been associated with protection in previous studies^2^. Another antigen candidate is apoB100, the carrier protein in LDL and modified versions of it^2, 4^. The possibilities are non-mutually exclusive. We here focus on MDA and anti-MDA.

MDA is highly reactive and forms protein adducts that are immunogenic as evidenced by recognition by antibodies. In addition, MDA itself can promote LDL-oxidation, and MDA-modified LDL is taken up by macrophages^5^. One example of an important modification caused by MDA is dihydropyridine (4-methyl-1,4-dihydropyridine-3,5-dicarbaldehyde) with the amino acid lysine. This stable compound is believed to play a role in atherosclerosis and other chronic inflammatory conditions^6^.

We here investigate a large prospective cardiovascular cohort of 60 years old men and women from Stockholm (60YO). We previously determined the role of IgM anti-MDA in this cohort and reported that it is associated with protection against CVD, especially among men^7^. We extend this study and investigate the role of some other isotypes and subclasses: IgG, IgG1 and IgG2 anti-MDA. The hypothesis was that high antibody levels are associated with protection and low levels with increased risk of disease, based on previous studies on other natural antibodies^2, 7^. The implications of the findings are discussed.

## Methods

### Subjects

The 60-year-old study has been described in detail elsewhere^8^. Briefly, from July 1st 1997, to June 30th, 1998, every third man and woman living in a part of the County of Stockholm, Sweden, reaching the age of 60 years, was invited to participate in a health screening for CVD. All in all, 4232 subjects (2039 men and 2193 women; response rate 78%) participated in the study. Information on sociodemographics, lifestyle habits, medication, and previous diseases and hospitalizations was obtained by a self-administered questionnaire. Physical examination was performed, including blood pressure measurements, anthropometry, and ECG. Serum, plasma, and whole blood were collected for storage in a biobank (−80 °C). The study was approved by the Karolinska Institutet research ethics committee and was carried out in accordance with the Declaration of Helsinki. All subjects gave informed consent before entering the study.

New coronary heart disease (CHD) events, were defined as fatal and non-fatal myocardial infarction (MI), ischemic stroke and hospitalization for angina pectoris. These were recorded as incident cases of the first CVD. The study base was matched with the national cause of death registry (fatal events until December 31, 2003) and with the national in-hospital registry (non-fatal events until December 31, 2005). Incident cases ( 211) of CVD were recorded and living subjects without a history of CVD before recruitment were included. The International Classification of Diseases (ICD-10) was used to register CHD-deaths (I 20, I 21, I 46), MI (I 21), angina pectoris including PCIs and CABGs (I 20, Z 95.5 and Z 95.1) and ischemic stroke (I 63-I 66). Three controls were randomly selected for each case and matched for gender and age (± 60 days). Thus, a nested case–control design (211 cases and 633 controls) was applied for the epidemiological and statistical analyses and 210 cases, and 620 controls were available for testing of the anti-MDA levels.

### Determination of antibodies against MDA with ELISA

#### Antibody determination

Anti-MDA IgG, IgG1, and IgG2 levels were measured by ELISA in essentially the same way as stated^9–12^. To ensure consistency between plates, Sigma's pooled serum was utilized. In each well, 10 μg/ml of antigen was coated with MDA-HSA to Nunc Immuno microwell plates from Thermo Labsystems in Franklin, MA. The coated plates were incubated at 4 degrees Celsius for 12 h. The plates were blocked with 2% BSA-PBS for 1 h at room temperature after being washed four times with wash buffer. After the same washing procedures, we diluted serum samples for IgG, IgG1, and IgG2 1:200 in 0.2% BSA-PBS and added them at 100 μl/well. After 2 h of incubation at room temperature and washing, as mentioned above, 100 μl/well of biotin-conjugated mouse antihuman IgG, mouse antihuman IgG1, and mouse antihuman IgG2 (diluted 1:80,000, 1:800, and 1:15,000 respectively in 1% BSA-PBS) were added. The plate was washed four times and then added with 100 μl/well of 1:5000 diluted horseradish peroxidase-conjugated streptavidin (0.2 percent BSA-PBS, Thermo Scientific, Denmark) for 20 min. TMB (3,30,5,50-tetramethylbenzidine; Sigma Aldrich, MO), a substrate for horseradish peroxidase, was added to the plates at a volume of 100 μl/well, and the plates were incubated for 10 min at room temperature in the dark to develop the color. The additional reaction was halted by adding 50 μl of 1N H_2_SO_4_ stop solution to each well. As a final step, we used an ELISA Multiscan Plus spectrophotometer (Spectra Max 250; Molecular Devices, CA) to analyze the plates at 450 and 540 nm for IgG, IgG1, and IgG2. The coefficient of variance for all the antibodies was less than 5% when all samples were evaluated twice in the same experiment.

#### Antibody specificity assay

Antibody specificity was tested by competition assay. The assay was performed according to the previous protocol^10^. In short, diluted sera were incubated with various concentrations of MDA-HSA (competitor). After vortexing, the sera with or without MDA-HSA incubation were tested for detection of IgG, IgG1, and IgG2 antibodies. Anti MDA antibody detection was inhibited above 70% in the sera with MDA-HSA (data not shown), demonstrating the specificity of the MDA antibody detection.

The inhibition was calculated as percentage; the formula as follows:

$$\% {\text{inhibition}} = \left( {{\text{OD}}\;{\text{without}}\;{\text{competitor}} - {\text{OD}}\;{\text{with}}\;{\text{competitor}}} \right)/{\text{OD}}\;{\text{without}}\;{\text{competitor}} \times 100.$$

### Statistical analysis

Various data analyses including demographic biochemistry- and anthropometry-related were performed for cases and controls respectively with values expressed as mean ± standard deviations (SD) for normally distributed parameters and medians (ranges) for parameters which were not normally distributed after logarithmic transformation. Statistical differences between cases and controls were evaluated through parametric tests. Odds ratios (OR) with 95% confidence intervals (CI) were calculated applying conditional logistic regression with anti-MDA levels divided into 7 percentiles as indicated. For the analyses of specific percentiles, the remaining values formed the reference. Analyses were run crude or adjusted for traditional risk factors as indicated. Correlation tests between CRP and antibodies was performed by Spearman test. These analyses were performed using SAS 9.4 release (SAS institute, Cary NC, USA).

## Results

### Clinical associations

We identified 211 incident cases of first CVD events throughout the follow-up period (77 with MI, 85 with angina pectoris, and 49 with ischemic stroke). For each incident case, three age and sex-matched controls were selected (633 controls in total). Serum samples were missing for 2 cases and 13 controls, leaving 209 cases and 620 controls for analyses.

As previously reported in a similar dataset^13^, which we still include here for clarity of presentation, there were more hypertensives and smokers among the cases than controls and a trendwise higher BMI. Blood pressure level, HDL, and hsCRP were associated with risk among cases as compared to controls (Table 1). Similar results were obtained for males and females separately (Tables 2 and 3). As presented in Tables 1, 2, 3, there were no significant differences in the median level of antibodies between groups, except that IgG1 Anti-MDA levels were lower among cases (median (interquartile range): 155.7(116.9–184.7) vs 162 (129.1–210.3); p = 0.039).

**Table 1:** Baseline characteristics among incident CVD cases and matched controls.

	Incident cases	Controls	p value
Number	209	620	NA
Age, years	60	60	NA
Male gender, %	66.0	66.8	NA
Smokers, % Combined	32.0	19.7	0.0002
Diabetes %	24.4	15.7	0.0042
BMI kg/m^2^	27.8 ± 4.6	26.6 ± 3.8	0.0030
Hypertension (> 140/90 mm Hg), %	42.6	25.7	< 0.0001
Glucose mmol/L	6.1 ± 2.5	5.6 ± 1.5	0.0004
Insulin µmol/L	11.4 ± 7.1	10.1 ± 59	0.0140
Systolic blood pressure, mm Hg	148 ± 21.8	139 ± 21.2	< 0.0001
Diastolic blood pressure, mm Hg	98 ± 10.6	85 ± 10.4	< 0.0001
Cholesterol, mMol/L	6.1 ± 1.0	6.0 ± 1.2	0.1366
HDL, mMol/L	1.3 ± 0.4	1.4 ± 0.4	0.0006
LDL, mMol/l	3.9 ± 1.2	3.8 ± 1.1	0.4490
Triglycerides, mMol/l	1.6 ± 1.0	1.4 ± 0.8	0.0003
hsCRP, mg/l	2.4 (1.3–4.6)	1.7 (0.9–3.2)	< .0001
Anti-MDA, IgG	136.5 (112.7–164.8)	133 (106.5–168.3)	0.44
antiMDA, IgG1	159.3 (126–192.9)	163 (130.5–209.9)	0.18
antiMDA, IgG2	122.4 (102.2–158.4)	128.9 (102.8–160.1)	0.57

hsCRP = high sensitivity C-reactive protein; MDA = Malondialedhyde; IgG = Immunoglobulin G. Comparisons between groups were made by use of Student’s T test for normally distributed values and by Kruskall-Wallis test for non-parametric variables.

**Table 2:** Baseline characteristics among incident CVD cases and matched controls among males.

	Incident cases	Controls	p value
Number	138	415	
Age, years	60	60	N/A
Smokers, %	32%	19%	0.0049
Diabetes %	29%	18%	0.0076
BMI kg/m^2^	27.7 ± 4.6	26.8 ± 3.6	0.0260
Hypertension (> 140/90 mm Hg), %	47%	30%	0.0003
Glucose mmol/L	6.2 ± 2.3	5.7 ± 1.6	0.0353
Insulin µmol/L	11.9 ± 7.9	10.4 ± 6.2	0.0427
Systolic blood pressure, mm Hg	149.4 ± 20.7	141.3 ± 20.8	< 0.0001
Diastolic blood pressure, mm Hg	90.8 ± 10.7	86.6 ± 10.4	< 0.0001
Cholesterol, mMol/L	6.0 ± 1.1	5.8 ± 1.0	0.0716
HDL, mMol/L	1.2 ± 0.3	1.3 ± 0.3	0.0073
LDL, mMol/l	3.9 ± 1.2	3.8 ± 1.0	0.4689
Triglycerides, mMol/l	1.7 ± 1.1	1.4 ± 0.9	0.0131
hsCRP, mg/l	2.5 (1.3–5.1)	1.7 (0.9–3.0)	< 0.0001
Anti-MDA, IgG	139.5 (110.8–166.5)	134.9 (109.1–170.3)	0.73
Anti-MDA, IgG1	155.7 (116.9–184.7)	162 (129.1–210.3)	**0.039**
Anti-MDA, IgG2	121.1 (100.5–154.7)	130 (104.4–161.8)	0.17

Significant values are in bold.

hsCRP = high sensitivity C-reactive protein; MDA = Malondialedhyde; IgG = Immunoglobulin G. Comparisons between groups were made by use of Student’s T test for normally distributed values and by Kruskall-Wallis test for non-parametric variables.

**Table 3:** Baseline characteristics among incident CVD cases and matched controls among females.

	Incident cases	Controls	p value
**Number**	71	205	
Age, years	60	60	N/A
Smokers, %	32%	20%	0.0482
Diabetes %	15%	11%	0.2820
BMI kg/m^2^	27.8 ± 4.8	26.4 ± 4.1	0.0260
Hypertension (> 140/90 mm Hg), %	32%	17%	0.0114
Glucose mmol/L	6.0 ± 2.7	5.4 ± 1.3	0.0754
Insulin µmol/L	10.3 ± 5.3	9.4 ± 5.9	0.1954
Systolic blood pressure, mm Hg	144.4 ± 23.8	133.7 ± 20.7	0.0004
Diastolic blood pressure, mm Hg	85.4 ± 9.9	81.0 ± 9.5	0.0008
Cholesterol, mMol/L	6.3 ± 1.0	6.2 ± 1.5	0.7728
HDL, mMol/L	1.5 ± 0.4	1.6 ± 0.4	0.0077
LDL, mMol/l	4.0 ± 1.2	3.9 ± 1.1	0.9819
Triglycerides, mMol/l	1.6 ± 0.9	1.2 ± 0.8	0.0018
hsCRP, mg/l	2.4 (1.4–4.4)	1.8 (1.0–3.4)	0.0150
Anti-MDA, IgG	134.6 (112.7–156.6)	126.3 (103.5–162.2)	0.35
Anti-MDA, IgG1	168.9 (133.8–206.2)	163.5 (132.7–208.5)	0.62
Anti-MDA, IgG2	123.2 (103.8–163.5)	123 (98.4–152.9)	0.33

hsCRP = high sensitivity C-reactive protein; MDA = Malondialedhyde; IgG = Immunoglobulin G. Comparisons between groups were made by use of Student’s T test for normally distributed values and by Kruskall-Wallis test for non-parametric variables.

anti-MDA levels were divided into percentiles, and low or high levels were compared with the rest as indicated (Table 4). After adjustment for smoking, BMI, type II diabetes, hypercholesterolemia, and hypertension, there were no significant differences for IgG anti-MDA. However, as indicated in Table 5, for IgG1 anti-MDA, a decreased risk was observed above 75th percentiles for CVD in general (0.69 (0.47–1.01)) and for MI/angina (0.58 (0.37–0.93)) while associations for stroke did not reach significance (data not shown). As indicated in Table 6, or IgG2 in the whole group (both men and women), there were no significant associations except above the 50th percentile which was associated with less risk of (0.70 (0.50–0.98)).

**Table 4:** Association between levels of IgG anti-MDA and risk for CVD, stroke, and MI.

Anti-MDA IgG	All		Males		Females	
	Crude	Adjusted	Crude	Adjusted	Crude	Adjusted
	OR (95% CI)		OR (95% CI)		OR (95% CI)	
Combined CVD as an outcome						
≤ 10%	0.78 (0.44–1.38)	8.88 (0.49–1.59)	0.81 (0.42–1.57)	0.87 /0.44–1.73)	0.69 (0.22–2.19)	0.78 (0.22–2.70)
≤ 25%	0.75 (0.51–1.11)	0.75 (0.50–1.12)	0.82 (0.51–1.33)	0.82 (0.50–1.35)	0.66 (0.35–1.25)	0.65 (0.33–1.27)
≤ 33%	0.83 (0.59–1.16)	0.83 (0.58–1.18)	0.98 (0.64–1.49)	0.98 (0.63–1.52)	0.60 (0.33–1.09)	0.60 (0.33–1.12)
> 50%	1.30 (0.94–1.79)	1.26 (0.89–1.76)	1.22 (0.82–1.81)	1.16 (0.77–1.75)	1.47 (0.83–2.59)	1.50 (0.81–2.75)
> 66%	1.12 (0.80–1.58)	1.03 (0.72–1.47)	1.29 (0.86–1.92)	1.15 (0.76–1.76)	0.79 (0.41–1.52)	0.77 (0.39–1.53)
> 75%	0.87 (0.59–1.25)	0.81 (0.55–1.19)	0.88 (0.57–1.37)	0.80 (0.50–1.27)	0.80 (0.40–1.62)	0.81 (0.39–1.68)
> 90%	0.69 (0.39–1.22)	064 (0.36–1.16)	0.69 (0.35–1.37)	0.63 (0.31–1.27)	0.68 (0.24–1.91)	0.68 (0.2 4–1.96)
Stroke as an outcome						
≤ 10%	0.97 (0.29–3.25)	1.49 (0.41–5.38)	1.16 (0.27–5.02)	2.33 (0.41–13.39)	0.69 (0.08–6.17)	0.90 (0.09–8.72)
≤ 25%	0.95 (0.45–2.01)	1.21 (0.55–2.68)	1.25 (0.43–3.63)	2.64 (0.69–10.12)	0.73 (0.25–2.16)	0.76 (0.24–2.43)
≤ 33%	1.09 (0.56–2.12)	1.36 (0.67–2.74)	1.59 (0.65–3.91)	3.26 (0.98–10.87)	0.69 (0.25–1.94)	0.72 (0.25–2.13)
> 50%	1.30 (0.67–2.53)	1.03 (0.49–2.14)	1.07 (0.45–2.55)	0.61 (0.21–1.81)	1.71 (0.60–4.87)	1.53 (0.47–5.04)
> 66%	0.69 (0.33–1.45)	0.58 (0.26–1.28)	0.52 (0.19–1.42)	0.34 (0.09–1.20)	1.00 (0.32–3.10)	0.91 (0.26–3.17)
> 75%	0.75 (0.34–1.65)	0.72 (0.31–1.64)	0.37 (0.10–1.33)	0.27 (0.06–1.20)	1.41 (0.47–4.22)	1.50 (0.47–4.81)
> 90%	0.45 (0.13–1.61)	0.62 (0.16–2.31)	0.30 (0.04–2.46)	0.41 (0.0 4–4.43)	0.62 (0.12–3.21)	0.71 (0.12–4.30)
MI/Angina as an outcome						
≤ 10%	0.73 (0.38–1.41)	0.83 (0.42–1.64)	0.75 (0.36–1.57)	0.80 (0.37–1.75)	0.69 (0.18–2.69)	0.65 (0.15–2.91)
≤ 25%	0.69 (0.44–1.09)	0.68 (0.43–1.08)	0.73 (0.43–1.27)	0.74 (0.42–1.30)	0.62 (0.28–1.37)	0.60 (0.26–1.38)
≤ 33%	0.75 (0.50–1.12)	0.75 (0.49–1.13)	0.86 (0.53–1.38)	0.85 (0.52–1.41)	0.57 (0.28–1.17)	0.58 (0.27–1.32)
> 50%	1.30 (0.89–1.88)	1.34 (0.90–1.98)	1.27 (0.81–1.97)	1.24 (0.78–1.97)	1.37 (0.69–2.71)	1.58 (0.75–3.32)
> 66%	1.28 (0.88–1.88)	1.20 (0.80–1.80)	**1.56 (1.00–2.42)**	1.41 (0.88–2.26)	0.71 (0.32–1.58)	0.70 (0.30–1.63)
> 75%	0.89 (0.58–1.37)	0.87 (0.55–1.36)	1.03 (0.64–1.65)	0.96 (0.58–1.59)	0.54 (0.20–1.42)	0.53 (0.19–1.48)
> 90%	0.78 (0.41–1.47)	0.71 (0.37–1.39)	0.79 (0.38–1.65)	0.69 (0.32–1.49)	0.73 (0.20–2.72)	0.70 (0.18–2.69)

Significant values are in bold.

Odds ratios (OR) with 95% confidence intervals (CI) were calculated applying conditional logistic regression.

**Table 5:** Association between levels of IgG1 anti-MDA and risk for CVD, stroke, and MI.

Anti-MDA IgG1	All		Males		Females	
	Crude	Adjusted	Crude	Adjusted	Crude	Adjusted
	OR (95% CI)		OR (95% CI)		OR (95% CI)	
Combined CVD as an outcome						
≤ 10%	1.21 (0.73–1.99)	1.20 (0.71–2.02)	**1.80 (1.01–3.23)**	**1.77 (0.96–3.26)**	0.37 (0.11–1.27)	0.40 (0.12–1.37)
≤ 25%	1.19 (0.83–1.71)	1.22 (0.83–1.77)	**1.48 (0.96–2.27)**	**1.53 (0.97–2.39)**	0.69 (0.34–1.41)	0.71 (0.34–1.48)
≤ 33%	1.13 (0.81–1.59)	1.19 (0.84–1.70)	1.23 (0.82–1.84)	1.31 (0.86–2.00)	0.94 (0.51–1.76)	1.00 (0.53–1.90)
> 50%	0.88 (0.64–1.21)	0.82 (0.59–1.14)	0.77 (0.52–1.15)	0.70 (0.47–1.06)	1.14 (0.66–1.98)	1.09 (0.61–1.94)
> 66%	**0.76 (0.54–1.07)**	**0.70 (0.49–1.01)**	**0.64 (0.41–0.99)**	**0.56 (0.36–0.89)**	1.03 (058–1.83)	1.00 (0.55–1.81)
> 75%	**0.69 (0.47–1.01)**	**0.59 (0.40–0.89)**	**0.58 (0.35–0.94)**	**0.46 (0.27–0.77)**	0.94 (0.50–1.78)	0.89 (0.46–1.72)
> 90%	0.82 (0.48–1.40)	0.70 (0.39–1.23)	0.90 (0.47–1.71)	0.71 (0.36–1.41)	0.66 (0.24–1.77)	0.67 (0.24–1.90)
Stroke as an outcome						
≤ 10%	0.92 (0.29–2.94)	1.08 (0.31–3.84)	1.88 (0.49–7.24)	4.32 (0.73–25.54)	N/A	N/A
≤ 25%	1.36 (0.66–2.83)	1.42 (0.66–3.09)	1.53 (0.62–3.82)	1.64 (0.56–4.84)	1.11 (0.32–3.80)	1.32 (0.37–4.73)
≤ 33%	1.15 (0.59–2.23)	1.33 (0.64–2.69)	0.94 (0.40–2.22)	0.92 (034–2.50)	1.56 (0.54–4.47)	1.87 (0.64–5.48)
> 50%	1.24 (0.63–2.46)	0.97 (0.46–2.04)	1.02 (0.41–2.53)	0.96 (0.32–2.86)	1.61 (0.57–4.56)	1.13 (0.37–3.40)
> 66%	0.82 (0.41–1.65)	0.76 (0.35–1.62)	0.44 (0.15–1.28)	0.33 (0.08–1.34)	1.51 (0.56–4.09)	1.29 (0.47–3.54)
> 75%	0.69 (0.32–1.49)	0.57 (0.24–1.35)	0.34 (0.10–1.22)	0.32 (0.07–1.42)	1.22 (0.45–3.33)	0.88 (0.29–2.66)
> 90%	1.00 (0.35–2.85)	0.78 (0.25–2.43)	0.35 (0.04–2.95)	0.25 (0.03–2.35)	1.79 (0.50–6.44)	1.20 (0.28–5.19)
MI/Angina as an outcome						
≤ 10%	1.29 (0.74–2.25)	1.40 (0.78–2.49)	1.79 (0.94–3.40)	1.95 (0.99.–3.84)	0.50 (0.14–1.79)	0.54 (0.15–1.95)
≤ 25%	1.14 (0.75–1.73)	1.19 (0.77–1.84)	1.46 (0.90–2.38)	1.57 (0.94–2.62)	0.56 (0.24–1.34)	0.56 (022–1.41)
≤ 33%	1.13 (0.76–1.67)	1.21 (0.80–1.82)	1.33 (0.83–2.11)	1.45 (0.90–2.35)	0.73 (0.34–1.58)	0.81 (035–1.85)
> 50%	0.80 (0.55–1.15)	0.76 (0.52–1.11)	0.72 (0.47–1.12)	0.66 (0.42–1.05)	0.99 (0.51–1.91)	0.97 (0.48–1.96)
> 66%	**0.74 (0.50–1.10)**	**0.66 (0.44–1.00)**	0.69 (0.43–1.12)	**0.56 (0.34–0.94)**	0.85 (0.42–1.73)	0.82 (0.39–1.75)
> 75%	**0.68 (0.44–1.07)**	**0.58 (0.37–0.93)**	0.64 (0.38–1.09)	**0.46 (0.26–0.82)**	0.80 (0.35–1.80)	0.88 (0.38–2.05)
> 90%	0.76 (0.41–1.43)	0.65 (0.34–1.27)	1.03 (0.52–2.04)	0.79 (0.38–1.65)	0.19 (0.02–1.43)	0.24 (0.03–1.91)

Significant values are in bold.

Odds ratios (OR) with 95% confidence intervals (CI) were calculated applying conditional logistic regression.

**Table 6:** Association between levels of IgG2 anti-MDA and risk for CVD, stroke, and MI among all participants and men and women.

Anti-MDA IgG2	ALL		Males		Females	
	Crude	Adjusted	Crude	Adjusted	Crude	Adjusted
	OR (95% CI)		OR (95% CI)		OR (95% CI)	
Combined CVD as an outcome						
≤ 10%	1.06 (0.62–1.79)	1.21 (0.70–2.09)	1.17 (0.61–2.25)	1.41 (0.72–2.78)	0.87 (0.35–2.17)	0.91 (0.36–2.33)
≤ 25%	1.01 (0.70–1.46)	1.07 (0.73–1.57)	1.25 (0.80–1.95)	1.39 (0.87–2.23)	0.67 (0.35–1.29)	0.66 (0.34–1.30)
≤ 33%	1.05 (0.75–1.47)	1.10 (0.78–1.58)	1.13 (0.74–1.72)	1.21 (0.78–1.88)	0.91 (0.51–1.61)	0.97 (0.53–1.78)
> 50%	0.77 (0.56–1.06)	**0.70 (0.50–0.98)**	0.71 (0.48–1.05)	**0.63 (0.41–0.95)**	0.89 (0.52–1.52)	0.85 (0.47–1.53)
> 66%	0.96 (0.69–1.35)	0.89 (0.62–1.26)	0.78 (0.51–1.19)	0.71 (0.4 6–1.10)	1.42 (0.81–2.50)	1.35 (0.74–2.49)
> 75%	0.92 (0.63–1.34)	0.84 (0.56–1.24)	0.76 (0.47–1.22)	0.66 (0.40–1.09)	1.30 (0.69–2.43)	1.32 (0.68–2.57)
> 90%	1.06 (0.64–1.77)	0.96 (0.56–1.63)	0.94 (0.50–1.76)	0.85 (0.44–1.65)	1.34 (0.57–3.12)	1.20 (0.48–3.00)
Stroke as an outcome						
≤ 10%	0.71 (0.19–2.64)	0.87 (0.23–3.31)	0.38 (0.04–3.41)	0.68 (0.07–7.12)	1.13 (0.22–5.84)	1.13 (0.20–6.39)
≤ 25%	0.55 (0.23–1.31)	0.52 (0.21–1.30)	0.53 (0.1 5–1.83)	0.71 (0.19–2.67)	0.57 (0.17–1.94)	0.44 (0.12–1.67)
≤ 33%	0.72 (0.35–1.51)	0.73 (0.33–1.58)	0.49 (0.14–1.48)	0.59 (0.17–2.04)	1.02 (0.40–2.63)	0.94 (0.33–2.70)
> 50%	0.63 (0.32–1.23)	0.63 (0.31–1.30)	0.66 (0.25–1.77)	0.63 (0.20–1.93)	0.60 (0.24–1.50)	0.54 (0.18–1.56)
> 66%	0.76 (0.37–1.54)	0.77 (0.35–1.67)	0.79 (0.29–2.16)	0.91 (0.28–2.95)	0.72 (0.26–2.00)	0.56 (0.17–1.81)
> 75%	0.57 (0.25–1.31)	0.57 (0.24–1.37)	0.64 (0.19–2.12)	0.71 (0.20–2.47)	0.51 (0.16–1.63)	0.32 (0.08–1.33)
> 90%	0.64 (0.20–2.03)	0.59 (0.17–2.12)	0.47 (0.05–4.19)	0.62 (0.05–7.36)	0.73 (0.18–2.92)	0.51 (0.10–2.56)
MI/Angina as an outcome						
≤ 10%	1.15 (0.64–2.06)	1.32 (0.72–2.41)	1.37 (0.69–2.73)	1.71 (0.82–3.55)	0.78 (0.26–3.33)	0.76 (0.25–2.34)
≤ 25%	1.18 (0.78–1.77)	1.30 (0.85–1.99)	1.45 (0.89–2.34)	**1.74 (1.04–2.91)**	0.72 (0.33–1.55)	0.76 (0.35–1.68)
≤ 33%	1.16 (0.79–1.70)	1.26 (0.84–1.89)	1.32 (0.84–2.08)	1.46 (0.90–2.36)	0.85 (0.42–1.73	1.00 (0.47–2.11)
> 50%	0.81 (0.56–1.17)	0.73 (0.50–1.08)	0.72 (0.47–1.11)	0.62 (0.39–0.99)	1.09 (0.56–2.11)	1.04 (0.51–2.13)
> 66%	1.03 (0.71–1.51)	0.91 (0.61–1.37)	0.78 (0.49–1.24)	0.67 (0.41–1.09)	1.99 (0.99–4.00)	1.96 (0.92–4.16)
> 75%	1.05 (0.69–1.61)	0.92 (0.58–1.45)	0.78 (047–1.32)	0.62 (0.36–1.08)	2.15 (0.97–4.74)	**2.57 (1.08–6.16)**
> 90%	1.22 (0.70–2.14)	1.10 (0.60–1.99)	1.02 (0.52–1.99)	0.89 (0.44–1.79)	2.02 (0.69–5.89)	2.19 (0.66–4.10)

Significant values are in bold.

Odds ratios (OR) with 95% confidence intervals (CI) were calculated applying conditional logistic regression.

If men were studied separately, IgG anti-MDA was not significantly associated with outcome when adjusted for covariates (Table 4). IgG1 anti-MDA was associated with protection in both CVD in general (0.56 (0.36–0.89)) 0.46 (0.27–0.77) and in MI/angina (0.56 (0.34–0.94)) 0.46 (0.26–0.82) while associations with stroke did not reach significance (Table 5). As indicated in Table 6, IgG2 anti-MDA above the median was associated with protection against CVD in the whole group and among men (0.70 (0.50–0.98); 0.63 (0.41–0.95) and with MI/angina among men (OR and 95% CI: 0.62; 0.39–0.99). However, among females, IgG2 anti-MDA above the 75th percentile instead was associated with an increased risk of MI/angina (OR and 95% CI: 2.57; (1.08–6.16)). Also, among men IgG2 anti-MDA were associated with protection, with low levels below 25th percentile having increased risk of MI/angina (1.74 (1.04–2.91)). Among women, studied separately, there were few significant associations with one notable exception: high IgG2 anti-MDA was associated with increased risk for development of MI/angina (2.57 (1.08–6.16)). Median values only differed significantly for IgG1 anti-MDA among men with lower levels among cases (p = 0.039).

We also studied associations with C-reactive protein and the antibodies studied. The associations reached significance for IgG (R = 0.11, p = 0.001) and IgG2 (R = 0.115, p < 0.001) but not for IgG1 (R = 0.05, p = 0.11) among all studied. We therefore also added CRP in the multivariate analyses, but with marginal effect, which did not influence the conclusions (Supplemental Table 1).

## Discussion

The main finding herein is that IgG1 antibodies against MDA conjugated with human albumin are associated with protection against CVD among 60-year-olds. This was apparent when high levels (above 66th and 75th percentile) were studied; while there were less pronounced differences at median levels, still median levels were higher among individuals who did not develop CVD during the follow-up time. Having high IgG1 anti-MDA levels was associated with having about half the risk of developing CVD. Similar associations were present also in relation to MI/angina, while associations with stroke did not reach significance. IgG2 anti-MDA was associated with protection against CVD and MI/angina in the whole group, and among men with MI/angina, but these associations were lower than for IgG1 anti-PC. In general, IgG anti-MDA had weak and non-significant associations with CVD protection.

There were interesting differences between men and women. In general, associations with protection were only present among men and strongest for IgG1 anti-MDA while not being present for IgG and for IgG2 anti-MDA only in the whole group and among men but weaker than IgG1 anti-MDA. Still, high levels of IgG2 anti-MDA, above the 75th percentile, were associated with increased risk of MI/angina among women.

We recently reported that IgM anti-MDA has a comparable association with CVD as IgG1 anti-MDA in this patient cohort, though somewhat stronger^7^. One difference is that IgM anti-MDA at low levels was associated with an increased risk of CVD (about double). Otherwise, associations were comparable for IgG1, the whole group, and men. There were no significant or non-significant associations or trends for IgM anti-MDA as a risk marker; high levels instead were associated with lower risk.

The finding that IgG1 (to some extent IgG2) and IgM anti-MDA is associated with protection in the whole group and among men could have interesting implications. It is in line with our recent observation that another so-called natural antibody, IgG1 anti-PC, is also a protection marker, albeit clearly stronger, in the same nested case–control study as here^14^. Further, the association with protection had a different profile for anti-PC IgG1, where low levels were associated with an increased risk of CVD among men as much as almost 10 times increased in relation to stroke. Also, IgG and IgG2 anti-PC showed some associations with increased risk at low levels. This contrasts with the present finding, where no such significant associations exist.

Also, the finding that high levels of IgG2 anti-MDA are associated with increased risk of MI/agina among women is somewhat unexpected and could also have interesting implications per se. Of note, it cannot be excluded that this is a chance subgroup finding and related to a relatively small number of women who develop CVD for 5 years follow-up time, at 60. Still, it cannot be excluded that anti-MDA, at least in some circumstances, can be a risk marker for forms of CVD, at least among women, as here. Among men, there was not even a trend in that direction; instead, OR was below 1.

One implication of this finding is that it may be advantageous, at least among men, to raise anti-MDA levels to protect against CVD. if so, the vaccine should be constructed to promote an IgM and IgG1 profile. Indeed, in line with this are animal studies where immunization with MDA-modified LDL is atheroprotective^15^.

Still, this finding among women could have interesting implications. If this finding can be confirmed, especially in larger studies, it may imply that anti-MDA could have some properties in common with anti-OxLDL antibodies, which have been reported to be both risk and protection markers in different studies, for reasons that are not clear. One may be the complexity of OxLDL as an antigen, and OxLDL cross-reacts with antiphospholipid antibodies (aPL), which are known to cause both arterial and venous thrombosis, especially in autoimmune and rheumatic conditions^16^.

In contrast to anti-MDA, we are not aware of any studies where IgG, IgG1, IgG2, or IgM anti-PC have been associated with increased risk, and especially IgG1 and IgM anti-PC are strong protection markers at high levels, which may even be stronger than an independent of known risk factors as smoking and hypertension^2, 4^.

The role of anti-OxLDL as a risk and/or protection marker has been studied relatively many times, with diverging results. There may be different non mutually exclusive causes of this. The methods of oxidation may differ, and methods of antibody determination. However, when we compared the two methods, the results were almost identical, and anti-OxLDL was associated with protection in borderline hypertension^3^. This finding is in line with important animal experiments where immunization with OxLDL ameliorated atherosclerosis development^17^.

In studies from the 90 s, anti-OxLDL was reported to be associated with atherosclerosis development, peripheral vascular disease, risk of MI, and coronary artery disease^18–22^. In line with this were studies in animal models where anti-OxLDL levels were raised in atherosclerotic mice^23^. There are also studies where no significant associations between anti-OxLDL and CVD were present^24–26^.

In other studies, the finding that anti-OxLDL can be associated with protection in relation to CVD has been confirmed^27–29^. On the other hand, a recent study implies that anti-OxLDL is associated with an increased risk of CVD and is also a marker of LDL oxidation^30^.

Instead of determining anti-OxLDL, focusing on different epitopes generated in this complex compound may be advantageous. In addition to MDA and PC, apoB100, the protein part of LDL, could also be of interest as an antigen. However, like anti-OxLDL, ApoB100, related antibodies have been associated with both protection^31^ and increased risk^32^.

Another interesting difference between PC and the other mentioned antigens is that PC is a pathogen-associated molecular pattern (PAMP) that is present on different pathogens and nematodes, parasites, and bacteria, functioning as a warning signal and eliciting a defensive immune reaction.

In addition, both PC and MDA are danger-associated molecular patterns exposed on dead cells and OxLDL^4^.

One reason anti-oxLDL maybe promote CVD in some circumstances could be that they cross-react with anti-cardiolipin antibodies aCL), which are known to be thrombogenic at high levels and cause both arterial and venous thrombosis^33^, especially among women and in autoimmune conditions as SLE and the antiphospholipid antibody syndrome^34^, while not being associated with atherosclerosis^35^. Interestingly, aCL is associated with CVD as MI also among non-autoimmune patients^36^. Since IgG2 anti-MDA indeed was associated with MI/angina among women at higher levels, the possibility that anti-MDA, under some circumstances, could behave as an aPL deserves further study. In general, antiphospholipid antibodies (aPL) are only present among a small proportion of the general population, 2,5% according to a recent study and mainly among women. Among these, only a minor proportion develop thrombosis or other aPL complications and this may occur through several different mechanisms^37^. This is in sharp contrast to the type of antibodies studied herein, which are present and detectable in all studied individuals according to studies we are aware of. Our present data indicate that IgG2 anti-MDA may promote a small increase in risk of CVD, but larger studies are needed to confirm this, and given the small numbers, it could also be a chance finding. Furthermore, if MDA could be the target of a vaccine to raise anti-MDA levels, this should be studied further, and a vaccine should be constructed to minimize IgG2 and promote IgG1 (and IgM) anti-MDA.

OxLDL is also known to form immune complexes, which could increase the risk of CVD; one is beta-2-Glycoprotein 1^38^. It is interesting to note that these can be involved in the thrombogenic properties of aCL and forms complexes with proteins such as beta-2 glycoprotein I with OxLDL, which induce T-cell activation^39^, so further studies on such MDA-complexes are also warranted.

Even though CRP has been reported to be associated with increased risk of CVD it is not clear if there is any causal association and which role CRP plays. It could be situation dependent. There were no significant associations between IgG1 anti-MDA and CRP but for IgG and IgG2 there were positive associations which reached significant. However, when controlled for, when significant, this did not affect the associations reported.

Even though underlying mechanisms for IgG1 anti-MDA which could provide an explanation to these associations are not so well known, IgM anti-MDA has interesting properties. We recently reported that IgM anti-MDA decreases oxidative stress and increases macrophage uptake of apoptotic cells^40^.

Limitations. One limitation is the size of the study. In a recent cross-sectional, much larger than previous studies, published data on IgM anti-MDA and also anti-PC where confirmed as associated with protection against MI (using two different methods)^41^ but this study was not prospective and did not include subclasses of these antibodies and the lack of such studies is a clear limitation. In such studies, the issue of potential adverse associations with outcome at very high levels (as those determined for aPL) could also be determined. Clearly larger prospective studies are needed to confirm these findings.

In conclusion, we here determine that IgG1, but not IgG and IgG2 anti-MDA are associated with protection against CVD and separately, MI/angina in the whole group and among men. This finding could improve risk evaluation and may also have therapeutic implications, e g by increasing the levels of these antibodies to protect against CVD through immunization. However, a caveat that needs further study is that IgG2 anti-MDA was a risk marker for MI/angina among women. These findings could still have implications for both prediction and therapy. In the future, increasing levels of anti-MDA through active (or passive) immunization could decrease risk of CVD. However, a caveat is IgG2 anti-MDA which, at least among women could constitute a problem if associations are confirmed in larger prospective studies).

### Supplementary Information

Supplementary Information.

## Data Availability

The datasets used and/or analyzed during the current study available from the corresponding author on reasonable request.
