# Supplementary material for: Antibodies against malondialdehyde among 60-year-olds: prediction of cardiovascular disease
Source: Sci Rep. 2023 Sep 11;13:15011. doi: 10.1038/s41598-023-42264-1 (PMC10495339; doi:10.1038/s41598-023-42264-1)
Supplement: Supplementary file 1 — Supplementary Information. [file 41598_2023_42264_MOESM1_ESM.docx]

**Supplemental table 1:** Association Between Levels of IgG anti-MDA or IgG2 anti-MDA and Risk for CVD, stroke, and MI among All Participants.

*adjusted: Model 2, as before.

**adjusted: adding crp, (log_scale) to model 2

Odds ratios (OR) with 95% confidence intervals (CI) were calculated applying conditional logistic regression

| **Combined CVD as an Outcome** | | | | | |
| --- | --- | --- | --- | --- | --- |
| **Cutoffs** | IgG anti-MDA | | IgG2 anti-MDA | |  |
|  | **Adjusted*** | **Adjusted**** | **Adjusted*** | **Adjusted**** |  |
|  | **OR (95% CI)** | | **OR (95% CI)** | |  |
| **< 10%** | 0.89 (0.49 – 1.59) | 0.85(0.47-1.55) | 1.21 (0.70 – 2.09) | 1.26 (0.73-2.19) |  |
| **< 25%** | 0.75 (0.50 – 1.12) | 0.78(0.52-1.17) | 1.07 (0.73 – 1.57) | 1.09 (0.74-1.61) |  |
| **< 33%** | 0.83 (0.58 – 1.18) | 0.86(0.60-1.23 | 1.10 (0.78 -1.58) | 1.16 (0.81-1.66) |  |
| **>50%** | 1.26(0.89–1.76) | 1.21(0.86-1.70 | **0.70 (0.50 – 0.98)** | **0.67 (0.48-0.95)** |  |
| **>66%** | 1.03 (0.72 – 1.47) | 0.97(0.67-1.39) | 0.89 (0.62 – 1.26) | 0.85 (0.59-1.21) |  |
| **>75%** | 0.81 (0.55 – 1.19) | 0.74(0.50-1.12) | 0.84 (0.56 – 1.24) | 0.80 (0.54-1.20) |  |
| **>90%** | 0.64 (0.36 – 1.16) | 0.61(0.34-1.11) | 0.96 (0.56 -1.63) | 0.90 (0.52-1.57) |  |
